# 胰岛素生长因子-1受体信号通路与肺癌的研究进展

**DOI:** 10.3779/j.issn.1009-3419.2010.06.014

**Published:** 2010-06-20

**Authors:** 文涛 岳

**Affiliations:** 101149 北京，北京市胸科医院 Department of Medical Oncology, Beijing Chest Hospital, Beijing 101149, Beijing

自1986年首次克隆了胰岛素生长因子- 1受体（insulin-like growth factor-1 receptor, IGF-IR）以后，人们围绕通过IGF-IR介导的经典的磷酸化激活的信号通路做了很多工作。在这条通路中，IGF-IR调节了细胞的增殖、运动、凋亡和死亡，使得我们又重新审视了生长激素（growth hormone, GH）-胰岛素样生长因子（insulinlike growth factor, IGF）-胰岛素样生长因子结合蛋白（insulin-like growth factor binding protein, IGFBP）轴在肿瘤发生发展中的作用。早在20多年前，人们就设想将胰岛素生长因子-1受体（IGF-IR）作为肿瘤治疗的靶点；随着近来对这一领域的进一步了解和相关临床试验的进行，有关GH-IGF-IGFBP轴在肿瘤中的作用得到了进一步明确。

## 1 IGF信号通路系统

IGF家族相对复杂，包括2个配体（IGF-I和IGFII）、2个受体（IGF-IR和IGF-IIR）、6个与IGF高亲和力的结合蛋白（IGFBP1-6）和其它与IGF低亲和力的IGF结合蛋白的相关蛋白（IGFBPrPs）^[1]^。在人类中，IGF-IRs和胰岛素受体在正常组织中广泛存在，每种受体都是四聚体的结构，具有两个“半受体”的特征，依次由主要在胞外与配体结合的α链和主要在胞内包括酪氨酸激酶区的β链组成。IGF-IIR并不转导信号传递，而是通过使IGF-II远离IGF-IR而降低IGF-II的活性，因而IGF-II具有抑癌基因的特性。

IGF-I和IGF-II对于IGF-IR有几乎相同的亲和力，但IGF-I和IGF-II对胰岛素受体有相对较低的亲和力^[2]^。IGFIR和胰岛素受体（InsR）共享近60%的同源氨基酸序列，特别是其激酶部分在氨基酸水平有近84%相同；IGF-IR可以与和它结构相似的胰岛素受体（InsR）形成混合体的形式存在，虽然他们可以同时接受IGF-I和胰岛素，但更优先接受IGF-I信号。IGF-IR在人类肿瘤细胞系中普遍表达，生理状态下的IGFs可以促使许多肿瘤细胞系产生有丝分裂^[3]^。IGF-IIR是单链的多肽，只与IGF-II结合，与IGF-IR不同，IGF-IIR无酪氨酸激酶的活性，而且它在IGF信号通路中的具体作用仍然不十分清楚。IGFBP通过调节IGF配体的生理活性和生物利用度来调控IGF的行为，这六种IGFBP可以通过分泌蛋白质来增强或抑制IGF的活性。

## 2 IGF-IR的生物学效应

胰岛素生长因子IGFs可以通过自分泌、旁分泌和内分泌的方式起作用。人类IGF-I和IGF-II的突变可以导致子宫内和出生后的发育不良、小头畸形和神经发育迟缓等症^[4]^。IGF-I除了是生长激素产生效应的主要传导介质外，也在细胞癌变的进程中起作用，包括细胞生长、分化、转化和作为凋亡的强抑制剂。IGF-IR系统还影响肿瘤细胞的运动、细胞密度和乏氧反应^[5]^。实际上，将IGF-IR作为研究靶点已经有近20年的时间，最初的体外研究显示了肿瘤细胞的增殖与IGF-I的浓度间存在剂量依赖的相关性，许多癌基因的转化活动需要IGF-I的信号传导系统^[6]^。IGF-IR在许多肿瘤细胞系中过表达，而抑制IGF-IR的表达不仅可以终止细胞的转化还可以逆转细胞的恶变，这不仅是与凋亡诱导下共同作用的结果，也是免疫系统对恶性细胞产生的反应^[7]^。IGF-I发挥其作用主要通过IGF-IR二聚体，也可以与IGF-IR及InsR的混合体结合进一步激活下游的信号，肿瘤细胞尤为如此。IGFs与IGF-IIR的结合不产生信号传导，所以，对IGFs来讲，IGF-IIR相当于它的洗涤槽^[8]^。

IGF-IR信号通路与其它生长因子的结合有利于启动肿瘤的发生发展，通过IGF-IR而激活其下游的PI3K-AKT信号通路，从而在肿瘤细胞内传递生存的信号，这可能是IGF轴与抗肿瘤治疗的原发或获得性耐药产生关联的原因。不同肿瘤细胞系和异种移植物给予IGF-I后会降低化放疗引起的细胞凋亡，而IGF-IR拮抗剂又被认为可以提高NSCLC细胞系对放疗的敏感性。

## 3 IGF-I介导的信号通路传导

非结合的IGF-I通过与位于细胞表面的IGF-IR的结合对糖类、脂类和蛋白质的代谢起主要作用^[9]^。与受体结合后，IGF-IR在经历了构象和自磷酸化后，通过胰岛素受体底物1-4（IRS1-4）和Src触发了胞内的级联反应，这些分子激活了IGF-IR下游的两个重要的信号转导通路：细胞分裂素活化蛋白激酶（MAPK）和磷脂酰激醇3激酶/AKT信号通路（PI3-K/AKT）^[10]^。MAPK信号通路的激活起始于Shc与IGF-IR近膜区域结合后导致的ERK的活化，沿着Ras/Raf/ERK/MAPK的下游区，活化的ERK1和ERK2引起了在转录和代谢方面的各种反应，从而影响细胞的生长、生存和分化。

PI3K信号通路的激活起始于胰岛素受体底物蛋白（IRS-proteins）和Shc的磷酸化，导致磷脂酰肌醇（3, 4, 5）三羟甲基氨基甲烷磷酸盐的增加，而PTEN可抑制上述反应。相应的下游的PDK1和AKT被激活后可影响细胞的生长、代谢、生存和细胞周期的延续。然后Ras/Raf和PI3K信号通路会汇聚在哺乳动物雷帕霉素靶点即mTOR上，同样可以影响细胞生长。

有许多证据^[11]^显示IGF-IR信号转导通路通过多种机制介导而涉及了肿瘤的发生、有丝分裂、转移、血管发生和抗凋亡等，并涉及了肿瘤化疗的耐药、放疗以及针对HER-2和EGFR的靶向治疗等方面。基础和临床试验也显示，同正常组织相比，IGF-IR在肿瘤组织中高表达。也有许多体外试验的结果^[12]^显示IGF-IR信号通路对其它信号通路有影响，如表皮生长因子（epidermal growth factor receptor, EGFR）和血管内皮生长因子（vascular endothial growth factor, VEGF）信号通路。在与EGFR信号通路的相互作用方面，IGF-IR的活化既可以引起EGFR通路的反式激活，同时也需要EGFR酪氨酸激酶的活性以激活下游的ERK通路，也可以直接的方式即EGFR与IGF-IR形成二聚物而引起ERK的蓄积^[13]^。目前已经明确IGF-IR与HER2/neu信号通路的相互作用。IGF-IR也可以与VEGF信号通路之间相互作用，VEGF的表达和分泌部分由IGFIR直接调节，PI3K/AKT和ERK/MAPK可增加VEGF的分泌，所以也间接地影响VEGF。有些研究^[14]^还报道了IGFIR与血小板衍生生长因子（platelet-derived growth factor, PDGF）之间的关系，在人成纤维细胞中，PDGF可以增加IGF-I的结合部位（如IGF-IR）。

## 4 胰岛素生长因子的靶向治疗对策

我们可以从多个层次上阻断IGF-IR信号转导通路。

首先，通过干扰生长激素释放激素以减少生长激素的产生，或使用生长激素的拮抗剂以减少血清中的IGF-I的水平。最近一种新型的生长激素受体拮抗剂培维索孟（pegvisomant）对IGF-I和IGF-II水平的抑制作用较奥曲肽更强，但仍需进一步的临床试验以研究其在IGF-I和IGFII依赖的肿瘤如乳腺癌和结直肠癌中的疗效^[15]^。另外，针对生长激素释放激素的拮抗剂JV-1-38已经完成动物实验，其对NSCLC细胞的治疗有效^[16]^。

其次，更多的对策是在研发IGF-IR和其下游的信号转导通路上的一些药物，如针对IGF-IR的单克隆抗体和酪氨酸激酶抑制剂。表 1列举了近期有关IGF、IGF-IR信号通路的在研新药。

**1:** 有关IGF、IGF-IR信号通路的在研新药物 New drugs related to IGF and IGF-IR signal pathway

Type	Target and compound	Company/manufacturer	Stage of development
Growth hormone releasing hormone antagonists			
	JV-1-38	Elixir	Preclinical
Growth hormone receptor antagonists			
	Pegvisomant	Pfizer	Preclinical
IGF-IR antibodies			
	CP-751 871	Pfizer	Phase Ⅰ/Ⅱ/Ⅲ
	AVE1642/EM164	Sanofi Aventis/ImmunoGen	Phase Ⅰ/Ⅱ
	IMC-A12	Imclone	Phase Ⅱ
	AMG-47, AMG-655	Amgen	Phase Ⅱ
	R1507	Roche	Phase Ⅰ/Ⅱ
MK-0646/h7C10	Merck/Pierre Fabre	Phase Ⅱ
	BIIBO22	Biogen Idec	Preclinical
	SCH-717454 (19D12)	Schering-Plough/Mederex	Phase Ⅰ/Ⅱ
IGF-1R antibody and IGF-1R TKI			
	NVP-AEW-541	Novartis	Preclinical
IGF-1R TKIs			
	BMS-536942	Bristol-Myers Squibb	Preclinical
	BMS-554417	Bristol-Myers Squibb	Preclinical
	NVP-ADW642	Novartis	Preclinical
	A-928605	Abbott	Preclinical
	AG1024	Calbiochem-EMDBiosciences	Preclinical
	OSI-906	OSI Pharmaceuticals	Phase Ⅰ/Ⅱ
	PQIP	OSI Pharmaceuticals	Preclinical
	PPP	Karolinska Institute/ Biovitrum	Preclinical
Dual inhibitor/ Antibody IGF-1R and EGFR			
	Di-diabody	Imclone	Preclinical
Dual inhibitor/ TKI IGF-1R and HER2 receptor			
	INSM-18/NDGA	Insmed	Phase Ⅰ/Ⅱ
Multitargeted inhibitor of IGF-1R, BCR-ABL, Src			
	XL-228/EXEL2280	Exelixis	Phase Ⅰ

## 5 针对IGF-IR的单克隆抗体

针对IGF-IR的单克隆抗体不仅可以阻断配体-受体相互作用，还可以下调受体反应。已经证实，IGF-IR的全激动药可以抑制肿瘤的生长，而且受体的下调作用比阻断配体-受体相互作用还要重要。目前已经开发出来的针对IGF-IR抗体的体内和体外实验均显示出广泛的抗肿瘤活性。

### 5.1 A12（IMC-A12，cixutumumab，Imclone公司）

这是一种完全人源化的IGF-IR的单克隆抗体，目前不仅可显著抑制肾癌、乳腺癌、胰腺癌的异种移植物的生长，同时在肿瘤切片的组织学研究中还发现，其可使凋亡细胞显著增加^[17]^。在已完成的每周给药的I期临床试验中，观察到2例疾病稳定（stable disease, SD）超过9个月的患者（男性乳腺癌和肝癌）。目前有超过18项的关于A12的临床试验正在进行中。

### 5.2 MK0646（Merck公司）

MK0646对乳腺、肺、大肠、头颈部、卵巢、胰腺、前列腺癌和黑色素瘤、神经细胞瘤、横纹肌肉瘤、骨肉瘤细胞系具有抑制作用。人源化的IGF-IR单抗h7C10/MK-0646体外实验显示，其对NSCLC细胞系A549具有明显的抑制作用，MK0646与化疗药长春瑞滨或EGFR单抗（西妥西单抗）联合时，几乎可完全抑制A549细胞的生长，而且动物实验显示MK-0646可以延长小鼠的生存，EGFR单抗与IGF-IR单抗联合组的生存优势更明显^[18]^。目前，有关MK-0646与西妥西单抗和伊立替康联合治疗曾接受过伊立替康治疗的转移性结直肠癌的Ⅱ/Ⅲ期临床试验正在进行。

### 5.3 AMG-479（Amgen, Thousand Oaks公司）

Ⅰ期临床试验表明，AMG-479在1例尤文氏肉瘤和1例神经源性肿瘤中显示了疗效，在随后进行的Ⅰb期临床试验中，AMG-479与帕尼单抗（panitumumab）或吉西他滨联合治疗实体瘤，AMG-479剂量为6 mg/kg-12 mg/kg，每2周给药；副作用包括高血糖、口腔炎及低镁血症等。1例部分缓解（partial relief, PR）患者是西妥西单抗治疗失败的K-RAS野生型的结肠癌患者，11例可评价的患者中9例为SD，帕尼单抗或吉西他滨并未影响AMG-479的药代动力学。

### 5.4 AVE-1642（sanofi-aventis公司）

该抗体的血浆半衰期约为9天，Ⅰ期临床试验探讨了第1周期3 mg/kg-18 mg/kg，每3周给药，第2周期始与多西紫杉醇75 mg/m^2^联合使用的疗效。结果显示，1例乳腺癌患者达到了PR，进一步的Ⅱ期临床试验正在进行中。

### 5.5 CP-751 871（figitumumab，Pfizer公司）

CP-751 871为另一种重要的人源化的IgG2抗体，其不仅具有良好的抗肿瘤活性同时也具有良好的药代动力学分布^[19]^。这些抗体与传统化疗药物如吉西他滨、长春瑞滨、阿霉素等联合时还显示出疗效的叠加作用。特别是2009年ASCO会议报道的CP-751 871与紫杉醇化疗方案联合治疗晚期转移性NSCLC的Ⅱ期临床试验的结果提示该药与传统化疗药物联合应用的前景。CP-751 871是完全人源化的免疫球蛋白单克隆抗体，半衰期为3周左右，Ⅰ期临床试验与标准剂量的紫杉醇和卡铂联用（PCI方案）治疗实体瘤时，未发生剂量限制性毒性反应，并表现出良好的耐受性^[20, 21]^。Ⅱ期临床试验按2:1的比例共入组患者156例，分别接受紫杉醇200 mg/m^2^、卡铂AUC6的静脉化疗，治疗组还接受CP-751 871 10 mg/kg或20 mg/kg（PCI_10_, PCI_20_）的IGF-IR单克隆抗体的治疗。在患者的人口学资料中，病理组织类型以腺癌为主，治疗组和对照组腺癌分别占48%和38%，其余为鳞癌和大细胞癌等；多数患者为吸烟患者，吸烟患者在两组分别占72%和66%。在无疾病进展的情况下，患者最多可接受6个周期的化疗，治疗组患者在化疗同时还会接受CP-751 871的治疗；对照组患者出现病情进展时也可接受CP-751 871的治疗。结果显示在肺鳞癌和腺癌患者中，两个剂量的治疗组PCI_10_、PCI_20_和对照组PC的有效率分别为33%、43%和62%；鳞癌患者接受PCI_20_治疗组的总体有效率[完全缓解（complete response, CR）+PR]为78%。值得注意的是CP-751 871的单药治疗对2例鳞癌患者同样有效。1例患者是对照组实施PC方案6周期化疗后出现疾病进展而后加用CP-751 871 10 mg/kg治疗；另1例患者是PCI_20_组在停用化疗后应用CP-751 871 20 mg/kg单药治疗，肿瘤继续缩小28%。与对照组的单纯PC方案化疗比较，PCI_20_组在无进展生存时间（progression-free survival, PFS）方面具有优势；有意思的是，与PC组相比，PCI_20_组中的非吸烟患者在PFS方面具有优势（7.27个月*vs* 3.08个月，因例数较少，统计学无差异）。在安全性方面，与CP-751 871相关的3、4级高血糖症在PCI组和PC组分别为15%和8%，PCI组中的3例患者因为高血糖症而终止治疗，采用胰岛素和口服降糖药物对症治疗。其它3、4级毒副反应，如中性粒细胞减少等血液学毒性和消化道反应等的发生率，两组类似。总之，IGF-IR单克隆抗体CP-751 871的Ⅱ期临床研究的结果证实其对局部晚期和转移性NSCLC患者的一线治疗的安全性和有效性，IGF-IR单克隆抗体可能会对NSCLC特别是肺鳞癌的治疗提供一个新的治疗选择，因为对现有的几种靶向治疗新药来讲，肺鳞癌患者并不是优势对象^[22]^。目前，前瞻性的Ⅲ期临床试验正在进行中，以验证上述的结果^[23]^。

## 6 针对IGF-IR信号通路下游的酪氨酸激酶抑制剂

正如上述所说，由于IGF-IR和胰岛素受体（InsR）高度的同源性，IGF-IR信号通路下游的酪氨酸激酶抑制剂与InsR酪氨酸激酶抑制剂间存在交叉抑制作用。很显然，针对此方面，药物的研发应集中发现对IGF-IR有高度选择性的酪氨酸激酶抑制剂。在体外研究^[24]^中发现，NVP-ADW742对一些实体瘤、血液肿瘤和多发性骨髓瘤细胞系显示了抗增殖活性和凋亡前期的反应。BMS-536924对乳腺癌MCF-7细胞系及其小鼠的异种移植物有活性。另外一个TKI——NVP-AEW541显示出对纤维肉瘤的活性，其药代动力学也符合其作为口服制剂的应用。

由于小分子IGF-IR抑制剂主要抑制IGF-IR、胰岛素受体和一些混合型受体，因此在抗肿瘤方面上较IGF-IR抗体更具活性。而且口服小分子抑制剂与其它药物联合使用时在续贯给药或间断给药等的最佳给药方式的选择上较IGF-IR抗体更加方便。早期结果揭示IGF-IR拮抗剂的安全性是可接受的，副作用也是可处理的。在使用IGF-IR抗体治疗的患者中，约1/4患者会出现轻度的血糖升高，因此推荐在治疗中监测患者血糖和脱水情况^[25]^。

由于目前肺癌治疗效果不理想，使得大家对肺癌治疗的关注较多，特别是IGF-IR信号通路和现有的EGFR TKIs之间有无关联以及能否克服抗EGFR治疗引起的获得性耐药等更是关注的焦点^[14]^。有研究^[26]^表明，IGF-IR的表达降低了针对EGFR的靶向治疗效果，对某些肺癌细胞系来说，AKT的活化被认为与EGFR TKIs的耐药有关。

部分患者可能是在服用EGFR TKIs的过程中与IGFIR形成EGFR-IGF-IR异源二聚体从而导致其下游信号的激活（如PI3K/Akt、p44/42 MAPK等）以及mTOR介导的蛋白的合成。近期有试验^[13]^表明抑制IGF-IR信号通路的酪氨酸激酶抑制剂AG1024联合厄洛替尼可以协同抑制肿瘤的生长。在另一项比较EGFR TKIs吉非替尼及抗EGFR的单克隆抗体西妥西单抗与AG1024联用的研究中显示吉非替尼与AG1024有协同作用，通过流式细胞仪检测的肺癌细胞系的结果显示，高水平的EGFR表达通常与IGF-IR相关，而且IGF-IR的表达水平可能决定了IGF-IR抑制剂的治疗效果^[27]^。然而，这一结论也有争议，另一项使用确诊时的标本进行免疫组化染色的回顾性研究^[28]^发现，IGF-IR的表达水平与疗效不相关，IGF-IR并未在吉非替尼原发耐药中起作用。

为探讨IGF-IR拮抗剂能否延长EGFR TKIs导致的继发耐药，一项随机的Ⅱ期临床试验正在进行中，该实验将IMC-A12（cixutumumab，完全人源化的IgG1抗体）与厄洛替尼联合使用治疗铂类耐药的晚期NSCLC患者，相关性的转译性研究也会同步进行^[29]^。其他的一些抗体如AMG-479、MK-0656以及小分子酪氨酸激酶抑制剂OSI-906等刚刚进入Ⅰ/Ⅱ期临床试验，几年后会有一些数据公布^[22]^。

## 7 今后的发展方向

在IGF-IR信号通路的转化性研究中，今后我们会更加关注以下几方面：

首先，针对IGF-IR的靶向治疗策略是否只针对IGFIR受体而与胰岛素受体IR间无或只有少部分交叉反应。正在研发的新化合物（如PPP）作为小分子酪氨酸激酶抑制剂，非ATP结合位点的竞争性抑制物也就自然地避免了与同一IR ATP结合区的交叉反应；PPP最近被证实下调IGF-IR而不影响IR，这在以前只在抗体中而不是在酪氨酸激酶抑制剂中观察到过。NVP-AEW-541就是一种靶点不仅作用在IGF-IR，还有其相关的酪氨酸激酶抑制剂的新药。

其次，一个值得关注的领域就是IGF-IR与其它信号通路间相互作用从而联合使用以达到治疗增益的作用，除与EGFR和HER2/neu信号通路拮抗剂合用并初步显示了治疗增益的希望以外，IGF-IR拮抗剂还有潜力与Ras/Raf/MEK/ERK或PI3K/Akt/mTOR信号通路的小分子拮抗剂联合使用，从而减少细胞增殖和促进凋亡。

第三，积极寻找预测治疗反应的标志物。目前的临床试验结果揭示了上皮来源（大多数鳞癌）或上皮-间质过度来源的肿瘤对IGF-IR拮抗剂治疗敏感，间质来源（未分化）的肿瘤对这类治疗反应不理想^[25]^。这也可以解释在CP-751 871与紫杉醇化疗药物联合治疗晚期转移性的NSCLC的Ⅱ期临床试验中鳞癌患者的总体有效率高达78%的结果；同时提示了应该根据不同受体和配体水平来选择不同预测标志物接受IGF-IR拮抗剂治疗的必要性和重要性。

总之，现有的临床数据支持短期和中期使用IGFIR拮抗剂的治疗，但长期接受IGF-IR拮抗剂的治疗是否可行以及现在就得出IGF-IR治疗肺癌有效性的结论还尚早；还需要在这一领域中做更多的工作和临床试验，以探讨IGF-IR与传统化疗药物和其他信号通路的阻断剂的最佳组合和最佳给药顺序以及该类药物最佳治疗人群和药物安全性。
